# Association between the frailty index and all-cause and cardiovascular mortality in a population with cardiovascular-kidney-metabolic syndrome: Insights from the NHANES 2011-2018

**DOI:** 10.1016/j.tjfa.2025.100131

**Published:** 2026-01-29

**Authors:** Xin Wang, Xinrui Hai, Ali Ma, Xiaolan Liang, Hua Cheng, Peng Wu, Yu Hao, Dapeng Chen, Ning Yan

**Affiliations:** a First Clinical College, Ningxia Medical University, Yinchuan, 750004, People’s Republic of China; b Heart Centre & Department of Cardiovascular Diseases, General Hospital of Ningxia Medical University, Yinchuan, 750004, People’s Republic of China; c Institute of Medical Sciences, General Hospital of Ningxia Medical University, Yinchuan, 750004, People’s Republic of China

**Keywords:** Cardiovascular-kidney-metabolic syndrome, Frailty index, All-cause mortality, Cardiovascular mortality

## Abstract

**Background:**

The Frailty Index (FI) is a well-established predictor of accelerated biological aging and a reliable tool for estimating all-cause and cardiovascular disease (CVD) mortality in older adults in the United States. However, its predictive value remains unclear in other U.S. population subgroups. This study aimed to examine the association between FI levels and both all-cause and CVD mortality among patients diagnosed with Cardiovascular-Kidney-Metabolic Syndrome (CKM syndrome).

**Methods:**

This study utilized the data from the National Health and Nutrition Examination Survey (NHANES 2011–2018), which included 7049 participants with complete information for CKM staging (stages 0–4). We employed multivariate Cox proportional hazards models in conjunction with restricted cubic splines (RCS) to account for potential non-linear relationships in the data. Additionally, segmented Cox proportional hazards models were used to examine the association between FI levels and both all-cause and CVD mortality in the CKM syndrome population. Subgroup analyses stratified by demographic and clinical factors, along with interaction tests, were performed to evaluate the consistency of these associations.

**Results:**

After adjusting for potential confounding variables, a nonlinear association was observed between the FI and CKM syndrome. Multivariable Cox regression analysis based on nationally representative data demonstrated that higher FI levels were significantly associated with increased risks of both all-cause and CVD mortality among patients with CKM syndrome. Multivariable analysis indicated a robust association between higher FI levels and the presence of CKM syndrome. Among patients diagnosed with CKM syndrome, each 10-unit increase in the FI was associated with a 54% higher risk of CVD mortality (HR = 1.54, 95% CI: 1.24–1.91; *P* < 0.001) and a 55% higher risk of all-cause mortality (HR = 1.55; 95% CI: 1.38–1.73, *P* < 0.0001). Stratified analyses revealed no significant interaction effects between the FI and demographic or clinical factors on mortality outcomes.

**Conclusion:**

The results highlight a robust and statistically significant association between FI and increased risk of both all-cause and CVD mortality among individuals with KM syndrome. Notably, FI may serve as a valuable marker for CKM stage stratification and for identifying high-risk patients.

## 1 Background

In an October 2023 scientific advisory, experts from the American Heart Association described CKM syndrome as a complex interactive pathophysiologic state involving metabolic dysfunction, CKD, and cardiovascular complications. This premise clearly articulates the interconnectivity among all conditions comprising the systemic disorder and underscores their collective impact on overall health. Therefore, the clinical course of this syndrome has been organized into a five-stage model that reflects its progressive clinical presentation, ultimately leading to multiorgan failure and a substantial increase in cardiovascular outcomes [1]. A significant amount of recent research has now firmly characterized CKM syndrome as a clinical entity. It therefore highlights the important, bidirectional interplay between metabolic dysfunction and the co-occurring conditions of chronic kidney disease (CKD) and cardiovascular disease [[2], [3], [4]]. More importantly, CKM syndrome constitutes an integrated pathophysiological state, where the interaction of its components synergistically elevates the risk of morbidity and mortality beyond the sum of their individual effects [5].

For example, the risk of developing heart failure is two to four times more among individuals with diabetes compared to those without diabetes [6], and the prevalence of CKD among people with diabetes approaches [7]. In the United States, about 5 % of adults are estimated to have cardiac, renal, and metabolic dysfunctions clustered, with prevalence rates increasing dramatically [8]. These observations suggest that CKM syndrome is more than the sum of its individual components. Rather, it reflects a more complex and synergistic disease process associated with substantially higher rates of mortality. By quantifying the accumulation of health deficits, the FI effectively captures the heterogeneity in biological aging across the adult lifespan. Individuals with similar chronological ages may have markedly different FI scores, which are strongly associated with adverse health outcomes [[9], [10], [11]].

Currently, the two most widely used definitions of frailty are the FI [12] and the Frailty Phenotype (FP) [13]. Studies using FI models demonstrate an exponential increase in the index with advancing age [14]; the FI not only predicts non-CVD mortality [15,16], but is also significantly associated with increased risk of CVD mortality and hospital readmissions [15,17,18]. Notably, the FI demonstrates predictive accuracy comparable to that of the Framingham Risk Score (FRS) for detecting cardiovascular events [19] and is applicable across all age groups above 20 years [14,20,21]. Frailty status, as measured by the FI or FP, is strongly associated with independent cardiovascular risk factors such as obesity and insulin resistance [20,[22], [23], [24], [25], [26]] and with poor cardiovascular health [27]. For instance, the impact of FI on the prognosis of heart failure extends beyond disease severity [28]. Furthermore, prospective cohort studies have shown that frailty is associated with an increased risk of developing cardiovascular disease and type 2 diabetes mellitus (T2DM). Evaluation for frailty could thus assist in identifying individuals at heightened risk for subsequent cardiovascular disease and type 2 diabetes, thereby facilitating the implementation of appropriate preventive and therapeutic interventions [29]. The most recent study by Farooqi et al. has demonstrated an association between the concurrent frailty and elevated CVD risk, as assessed by the FRS, as well as with incident cardiovascular disease and related mortality [19].

Currently, the association between the FI and all-cause as well as CVD mortality among individuals with CKM syndrome remains unclear, and no study has yet examined the prognostic value of FI in the CKM population defined by the updated AHA criteria. This study is clinically important because CKM syndrome occupies a central position in the continuum of cardiovascular events and may lead to substantial adverse health outcomes [30,31]. Therefore, this study aims to evaluate the relationship between FI and mortality risk in CKM syndrome, thereby informing frailty-based risk stratification and facilitating the development of early warning strategies to reduce the burden of cardiovascular disease through lifestyle interventions and metabolic control [1].

## 2 Methods

### 2.1 Study population

**Fig. 1:**
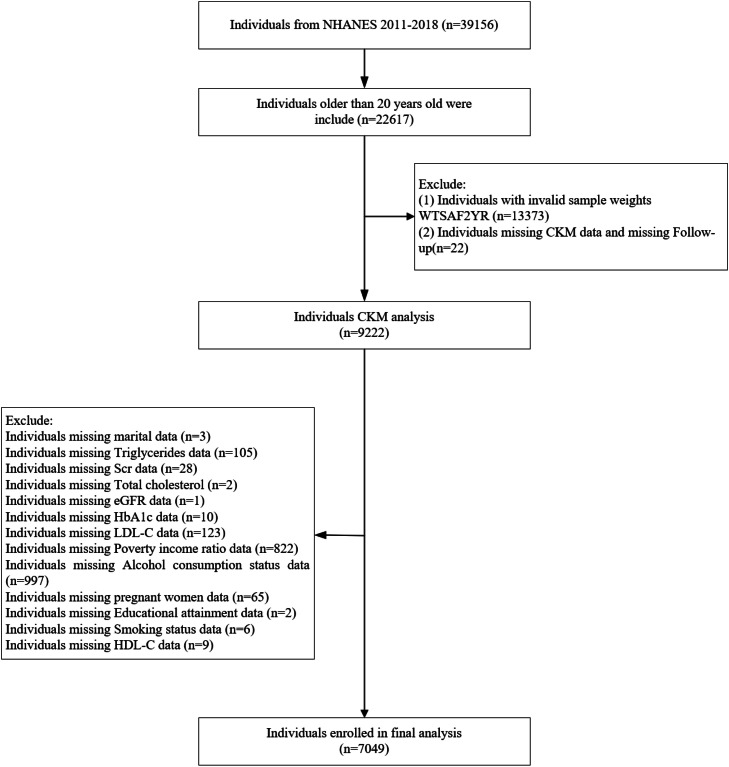
Flowchart of the study population Abbreviations: NHANES, National Health and Nutrition Examination Survey; CKM, Cardiovascular-Kidney-Metabolic Syndrome.

An analysis was conducted on data from four consecutive cycles (2011–2018) of the National Health and Nutrition Examination Survey (NHANES). NHANES is a comprehensive, cross-sectional study administered by the National Center for Health Statistics. The NHANES data constitute a robust database for assessing the health and nutritional status of the noninstitutionalized civilian population of the United States. The survey has been conducted with careful attention as far as sampling is concerned; it is multistage and probabilistic, aimed at achieving a study sample representative of the U.S. population. This study used publicly available datasets provided by the Centers for Disease Control and Prevention (http://www.cdc.gov/nchs/nhanes.htm). We started with 39,156 participants and excluded: (1) Participants aged < 20 years (*n* = 16,539), (2) Missing CKM syndrome parameters and follow-up data (*n* = 22), (3) Pregnant individuals (*n* = 65), (4) Incomplete covariate information (*n* = 2108), (5) Individuals missing marital data (*n* = 3), (6) Individuals missing triglycerides data (*n* = 105), (7)Individuals missing serum creatinine (Scr) data (*n* = 28), (8) Individuals missing total cholesterol data (*n* = 2), (9)Individuals missing eGFR data (*n* = 1), (10) Individuals missing HbA1c data (*n* = 10), (11) Individuals missing LDL-C data (*n* = 123), (12) Individuals missing poverty income ratio data (*n* = 822), (13) Individuals missing alcohol consumption status data (*n* = 997), (14)Individuals missing educational attainment data (*n* = 2), (15)Individuals missing smoking status data (*n* = 6), (16)Individuals missing HDL-C data (*n* = 9). After applying these criteria, the final analytic sample comprised 7049 eligible participants. The flowchart of the study population selection is presented in Fig. 1. Prior to participating in the survey, all participants provided written informed consent. The NHANES study protocol was reviewed and approved by the Ethics Review Committee of the National Center for Health Statistics, and this approval was renewed for each subsequent survey cycle.

### 2.2 Measurement of FI

The FI was calculated according to the methodology described by Searle et al [32]. The present study used a multisystem approach to assess health deficits. A total of 53 deficits were identified across seven physiological domains. These domains include cognitive function, mood disturbances, degree of dependence, coexisting medical conditions, health services utilization, physical functioning, and laboratory data. The cognitive function domain includes one indicator, whereas mood disturbances are measured using seven indicators. The degree of dependence is assessed using 20 criteria. Coexisting medical conditions are represented by 13 items, and health services utilization and accessibility are measured by five indicators. Physical functioning and body measurements are assessed with one indicator, and laboratory data are represented by six variables. Each component in the FI is scored on a scale from 0 to 1, reflecting the degree of dysfunction. For a detailed list of the variables included in the FI, refer to the supplementary table (Supplementary Table S1). A more exhaustive explanation of the index calculation is found elsewhere [[32], [33], [34]].

### 2.3 Definitions of CKM syndrome stages 0 to 4

CKM syndrome is defined as a multiorgan disorder, characterized by interdependent dysfunction across the cardiac, renal, and metabolic systems [1]. We categorized CKM syndrome into five escalating severity levels (denoted as Stages 0 through 4), according to the criteria from both AHA and Aggarwal et al [1,35]. Stage 0: No identified CKM risk factors; with metabolic parameters (including blood glucose and blood pressure) maintained within normal physiological ranges. Stage 1: Presence of adiposity-related abnormalities, specifically obesity (BMI ≥ 30kg/m²), excess body fat distribution, or biochemical evidence of adipose tissue dysfunction. Stage 2: Existence of Metabolic Risk Indicators (hypertension, dyslipidemia, or impaired glucose metabolism) and/or diagnosis of CKD with intermediate to high renal risk (based on KDIGO criteria). Stage 3: Subclinical CVD is defined as estimated 10-year atherosclerotic CVD risk ≥20 %, as determined by the fundamental PREVENT formula [36]. "Very high-risk CKD" referred to stages 4 or 5 CKD. The base equations can be found in Supplementary Material 1, Table S1. Stage 4: Clinically evident CVD in the context of: CKM syndrome, adiposity disorders, or metabolic/CKD risk profiles. CKD is categorized based on the KDIGO guidelines, which base assessment primarily on estimated glomerular filtration rate (eGFR) and urine albumin-to-creatinine ratio (UACR) [37]. For this assessment, eGFR values were calculated using the race-neutral 2021 CKD-EPI formula [38]. Comprehensive staging criteria are detailed in Supplementary Material, Table S2.

### 2.4 Assessments of other covariates

Data on demographic characteristics, socioeconomic status, lifestyle behaviors, and clinical comorbidities were obtained through standardized in-home interviews and assessments at the mobile examination center (MEC). Demographic and socioeconomic covariates included age, gender, race/ethnicity (categorized as non-Hispanic white, non-Hispanic black, Mexican American, or other races), marital status, educational attainment, and the poverty income ratio (PIR). Lifestyle risk factors were assessed based on body mass index (BMI), smoking status, and alcohol consumption, with participants categorized into never, former, or current users according to lifetime usage patterns. Furthermore, the presence of key comorbidities, specifically hypertension, diabetes mellitus, and hyperlipidemia, was ascertained through a rigorous combination of self-reported medical history, medication usage, and laboratory testing (including blood pressure, fasting glucose, HbA1c, and lipid panels). Specific diagnostic criteria and detailed definitions for all covariates are summarized in Supplementary Table S3. These criteria are consistent with established clinical guidelines for the diagnosis of these conditions [39].

### 2.5 Statistical analysis

This study followed the analytical protocols for the NHANES 2011–2018 complex survey design. To account for the complex survey design, strata, primary sampling units, and sample weights were incorporated. Continuous variables are presented as mean ± standard deviation (SD) when normally distributed and median with interquartile range (IQR) when non-normally distributed variables. Categorical data are presented as frequency counts and percentage rates. Participants were categorized into two cohorts based on the FI, with the lower-frailty cohort serving as the reference group. The optimal FI cutoff for this stratification was determined using receiver operating characteristic (ROC) curve analysis to enhance clinical application potential [40]. In accordance with the STROBE guidelines [41], we evaluated the association between FI and both all-cause mortality and CVD mortality among individuals with CKM syndrome. Multivariate linear regression was used to quantify the association between FI and progressive CKM syndrome staging (0–4). Our analysis encompassed three hierarchical models: Model I: Unadjusted. Model II: Adjusted for demographic factors (age, sex, race/ethnicity, marital status, education level), behavioral factors (smoking status, alcohol consumption status), and socioeconomic status (poverty income ratio [PIR]). Model III: Model II + further adjusted for clinical biomarkers (lipid measures: triglycerides [TG], total cholesterol [TC], high-density lipoprotein cholesterol [HDL-C]; low-density lipoprotein cholesterol [LDL-C], hematological parameters: platelet count [PLT], hemoglobin [HGB]; renal function markers: serum creatinine [Scr], estimated glomerular filtration rate [eGFR], uric acid) and clinically diagnosed comorbidities (hypertension, hyperlipidemia, diabetes mellitus). To examine the threshold effect of the FI on all-cause and CVD mortality, we employed segmented linear regression. Potential nonlinear associations between FI and mortality outcomes were further explored using RCS functions within the multivariable Cox models. The dose-response relationship between CKM syndrome staging and the risk of mortality (all-cause or CVD) was examined using a RCS model. This analysis spanned the full range of CKM stages, from 0 to 4, and included a comparative assessment between the early stages (0–2) and the late stages (3**–**4). To strengthen the credibility of the findings, the study systematically assessed the demographic differences between populations with varying FI levels and those with CKM syndrome using complementary approaches, including subgroup analysis. This study also evaluated the discriminatory ability of FI across CKM stages 0–4 and for mortality outcomes. All-cause and CVD mortality were analyzed using ROC curve analysis, with performance assessed by the area under the curve (AUC). Survival probabilities were estimated using Kaplan-Meier curves, and mortality outcomes were compared between FI groups. The optimal FI cutoff was determined through ROC analysis based on the Youden index. A statistical comparison between the groups was performed using the log-rank test. All statistical analyses were performed using EmpowerStats version 4.2 (www.empowerstats.com, X&Y solutions, Inc. Boston MA)

## 3 Results

### 3.1 Baseline characteristics of participants and outcome parameters

Table 1 presents the baseline characteristics of the study participants, classified by CKM syndrome subsets. The flowchart for this study sample is shown in Fig. 1. The study population included 7049 individuals with a mean age of 49.49 years; 3490 (49.51 %) were men. The all-cause mortality rate was 6.14 % (433 / 7049), CVD mortality rate was 1.67 % (118 / 7049), At baseline, the mortality rate among participants in CKM stage 3 was higher than that among those in stage 4. Significant differences across CKM stages were observed for gender, age, race, BMI, educational attainment, alcohol consumption, smoking status, hypertension, hyperlipidemia, diabetes prevalence and the distribution of the PIR.

**Table 1:** Baseline characteristics of individuals classified by CKM staging.

Characteristic		Overall			Cardiovascular-Kidney-Metabolic syndrome																	P-value	
					0, N = 555		1, N = 1473				2, N = 3842			3, N = 399			4, N = 780						
Median																							
AGE		49.49 ± 17.47			33.09 ± 12.36		39.86 ± 14.17				49.66 ± 15.15			74.22 ± 8.62			65.86 ± 12.44					<0.001	
Gender ( %)																						<0.001	
Male		3490 (49.51 %)			197 (35.50 %)		675 (45.82 %)				1918 (49.92 %)			264 (66.17 %)			436 (55.90 %)						
Female		3559 (50.49 %)			358 (64.50 %)		798 (54.18 %)				1924 (50.08 %)			135 (33.83 %)			344 (44.10 %)						
PIR		2.54 ± 1.63			2.73 ± 1.66		2.69 ± 1.66				2.55 ± 1.64			2.26 ± 1.50			2.23 ± 1.49					<0.001	
BMI		29.35 ± 7.18			21.44 ± 2.07		28.12 ± 5.59				30.73 ± 7.37			29.09 ± 6.34			30.66 ± 7.61					<0.001	
Race ( %)																						<0.001	
Non-Hispanic White		2854 (40.49 %)			259 (46.67 %)		555 (37.68 %)				1436 (37.38 %)			187 (46.87 %)			417 (53.46 %)						
Non-Hispanic Black		1434 (20.34 %)			72 (12.97 %)		273 (18.53 %)				828 (21.55 %)			94 (23.56 %)			167 (21.41 %)						
Mexican		953 (13.52 %)			39 (7.03 %)		226 (15.34 %)				580 (15.10 %)			39 (9.77 %)			69 (8.85 %)						
Other Hispanic		1808 (25.65 %)			185 (33.33 %)		419 (28.45 %)				998 (25.98 %)			79 (19.80 %)			127 (16.28 %)						
Education ( %)																						<0.001	
Some college or above		5639 (80.00 %)			500 (90.09 %)		1246 (84.59 %)				3040 (79.13 %)			282 (70.68 %)			571 (73.21 %)						
High School		857 (12.16 %)			41 (7.39 %)		144 (9.78 %)				499 (12.99 %)			55 (13.78 %)			118 (15.13 %)						
Middle School		553 (7.85 %)			14 (2.52 %)		83 (5.63 %)				303 (7.89 %)			62 (15.54 %)			91 (11.67 %)						
CVD ( %)																						<0.001	
No		6292 (89.26 %)			555 (100.00 %)		1473 (100.00 %)				3842 (100.00 %)			399 (100.00 %)			23 (2.95 %)						
Yes		757 (10.74 %)			0 (0.00 %)		0 (0.00 %)				0 (0.00 %)			0 (0.00 %)			757 (97.05 %)						
CVD mortality ( %)																				<0.001			
No		6931 (98.33 %)			554 (99.82 %)		1469 (99.73 %)			3818 (99.38 %)				371 (92.98 %)			719 (92.18 %)						

Characteristic			Overall			Cardiovascular-Kidney-Metabolic syndrome																P-value	
						0, N = 555			1, N = 1473				2, N = 3842			3, N = 399		4, N = 780					
Yes			118 (1.67 %)			1 (0.18 %)			4 (0.27 %)				24 (0.62 %)			28 (7.02 %)		61 (7.82 %)					
all-cause mortality ( %)																						<0.001	
No			6616 (93.86 %)			549 (98.92 %)			1456 (98.85 %)				3702 (96.36 %)			293 (73.43 %)		616 (78.97 %)					
Yes			433 (6.14 %)			6 (1.08 %)			17 (1.15 %)				140 (3.64 %)			106 (26.57 %)		164 (21.03 %)					
Diabetes ( %)																						<0.001	
No			5547 (78.69 %)			555 (100.00 %)			1473 (100.00 %)				2934 (76.37 %)			161 (40.35 %)		424 (54.36 %)					
Yes			1502 (21.31 %)			0 (0.00 %)			0 (0.00 %)				908 (23.63 %)			238 (59.65 %)		356 (45.64 %)					
Hyperlipidemia ( %)																						<0.001	
No			2113 (29.98 %)			384 (69.19 %)			749 (50.85 %)				806 (20.98 %)			91 (22.81 %)		83 (10.64 %)					
Yes			4936 (70.02 %)			171 (30.81 %)			724 (49.15 %)				3036 (79.02 %)			308 (77.19 %)		697 (89.36 %)					
Hypertension ( %)																						<0.001	
No			4026 (57.11 %)			555 (100.00 %)			1473 (100.00 %)				1735 (45.16 %)			77 (19.30 %)		186 (23.85 %)					
Yes			3023 (42.89 %)			0 (0.00 %)			0 (0.00 %)				2107 (54.84 %)			322 (80.70 %)		594 (76.15 %)					
Marital status ( %)																						<0.001	
Widowed/Divorced/Never married			2848 (40.40 %)			292 (52.61 %)			560 (38.02 %)				1487 (38.70 %)			174 (43.61 %)		335 (42.95 %)					
Married/Living with Partner			4201 (59.60 %)			263 (47.39 %)			913 (61.98 %)				2355 (61.30 %)			225 (56.39 %)		445 (57.05 %)					
Smoke ( %)																						<0.001	
Never			3983 (56.50 %)			397 (71.53 %)			929 (63.07 %)				2160 (56.22 %)			181 (45.36 %)		316 (40.51 %)					
Current			1368 (19.41 %)			93 (16.76 %)			250 (16.97 %)				778 (20.25 %)			69 (17.29 %)		178 (22.82 %)					
Former			1698 (24.09 %)			65 (11.71 %)			294 (19.96 %)				904 (23.53 %)			149 (37.34 %)		286 (36.67 %)					
Alcohol ( %)																							

Characteristic	Overall			Cardiovascular-Kidney-Metabolic syndrome																		P-value	
				0, N = 555				1, N = 1473				2, N = 3842			3, N = 399				4, N = 780				
Never	979 (13.89 %)			76 (13.69 %)				181 (12.29 %)				528 (13.74 %)			81 (20.30 %)				113 (14.49 %)				
Current	5130 (72.78 %)			446 (80.36 %)				1178 (79.97 %)				2818 (73.35 %)			223 (55.89 %)				465 (59.62 %)				
Former	940 (13.34 %)			33 (5.95 %)				114 (7.74 %)				496 (12.91 %)			95 (23.81 %)				202 (25.90 %)				
BUN (mg/dL)	4.94 ± 2.05			4.24 ± 1.34				4.51 ± 1.42				4.70 ± 1.55			7.21 ± 3.70				6.24 ± 2.97			<0.001	
eGFR (mL/min/1.73 m²)	94.92 ± 23.27			109.19 ± 17.74				104.43 ± 18.03				96.75 ± 19.75			62.78 ± 23.15				74.23 ± 23.90			<0.001	
HDL-C (mg/dL)	1.40 ± 0.41			1.62 ± 0.40				1.49 ± 0.36				1.35 ± 0.41			1.37 ± 0.40				1.34 ± 0.43			<0.001	
Hemoglobin (g/dL)	14.10 ± 1.53			14.02 ± 1.30				14.06 ± 1.46				14.24 ± 1.52			13.63 ± 1.79				13.85 ± 1.64			<0.001	
LDL-C (mg/dL)	2.90 ± 0.91			2.55 ± 0.73				2.92 ± 0.81				3.04 ± 0.94			2.76 ± 0.88				2.56 ± 0.96			<0.001	
Platelet	235.05 ± 61.72			225.63 ± 47.80				235.73 ± 56.44				241.24 ± 63.30			216.11 ± 66.98				219.67 ± 64.05			<0.001	
Scr (mg/dL)	79.05 ± 43.97			70.28 ± 15.02				72.70 ± 16.46				74.49 ± 17.77			124.75 ± 140.70				96.36 ± 59.55			<0.001	
Total cholesterol (mg/dL)	4.91 ± 1.06			4.57 ± 0.87				4.83 ± 0.92				5.08 ± 1.08			4.75 ± 1.04				4.51 ± 1.14			<0.001	
Triglycerides (mg/dL)	1.26 ± 0.74			0.74 ± 0.28				0.86 ± 0.31				1.47 ± 0.80			1.31 ± 0.68				1.37 ± 0.74			<0.001	
Uric acid (mg/dL)	327.26 ± 84.46			275.98 ± 64.45				303.84 ± 74.11				334.31 ± 82.18			363.41 ± 95.36				354.82 ± 93.15			<0.001	
WC (cm)	99.71 ± 16.78			78.11 ± 6.53				94.86 ± 12.79				103.07 ± 16.51			104.21 ± 14.73				106.10 ± 16.32		<0.001		
Frailty index	0.15 ± 0.09			0.09 ± 0.05				0.10 ± 0.06				0.14 ± 0.08			0.20 ± 0.09				0.25 ± 0.10		<0.001		

Abbreviations: BMI, Body Mass Index; PIR, Poverty income ratio; CVD, Cardiovascular Disease; CKD, Chronic Kidney Disease; LDL-C, Low-Density Lipoprotein Cholesterol; Scr, Serum Creatinine; HDL-C, High-Density Lipoprotein Cholesterol; eGFR, Estimated Glomerular Filtration Rate. Continuous variables with normal distributions are presented as mean ± standard deviation (SD), while non-normally distributed continuous variables were described using medians and interquartile ranges (IQRs). Categorical variables are summarized as frequencies and percentages.

### 3.2 Association of FI and CKM syndrome

After adjusting for potential confounders, the analyses revealed a nonlinear association between the FI and CKM syndrome using RCS regression (Fig. S1). The optimal FI cutoff was determined to be 0.1512 through ROC analysis based on the Youden index [40]. Using the low-FI group (FI ≤ 0.1512) as the reference, the high-FI group showed significantly higher odds of CKM syndrome, with a ratio (OR) of 2.56 (95 % CI:2.19**–**3.00; *P* < 0.0001) (Table 2). These findings indicate that individuals with FI values above 0.1512 are at significantly increased risk of CKM syndrome. The FI demonstrates clinical relevance as a risk-stratification marker among CKM syndrome patients.

**Table 2:** Levels of frailty index in relation to CKM Stage 0 to 4.

	Unadjusted			Adjust I		Adjust II	
	HR (95 %CI)		P	HR (95 %CI)	P	HR (95 %CI)	P
Two-piecewise logistic regression model (Inflection point = 0.1512)							
FI ≤0.1512	1.0			1.0		1.0	
FI > 0.1512	6.34(5.51,7.30)	<0.0001		3.26 (2.81, 3.77)	<0.0001	2.56 (2.19, 3.00)	<0.0001
Binary Variable (Cut-off point = 0.18)							
FI ≤ 0.18	1.0			1.0		1.0	
FI >0.18	5.18 (4.58, 5.86)	<0.0001		2.75 (2.42, 3.13)	<0.0001	2.14(1.86, 2.46)	<0.0001

Abbreviations: HR, hazard ratio; CI, confidence interval; CKM syndrome, Cardiovascular-Kidney-Metabolic Syndrome; FI, Frailty index. Unadjusted model adjusted for: None.

Model I was adjusted for: Gender, Age, Race, Marital status categorized, PIR, Educational level, Alcohol consumption categorized, Smoking status.

Model II was adjusted for: Gender, Age, Race, Marital status categorized, PIR, Educational level, Smoking status, Alcohol consumption categorized, Scr, UA, TG, TC, HDL-C, LDL-C, Hypertension, Diabetes, Hyperlipidemia, PLT, eGFR, HGB.

### 3.3 Associations between FI and all-cause and CVD mortality

Table 3 presents the association between the frailty index (FI) and mortality rates due to all causes and CVD diseases. The table illustrates the association of the FI with the probability of all-cause and CVD mortality across stages 0–4 of CKM syndrome. Model I, with the low FI group serving as the baseline, revealed a 158 % higher risk of CVD mortality with each 10-point increase in the FI (HR = 2.58; 95 % CI:2.20**–**3.03, *P* < 0.0001). There was a 131 % increase in all-cause mortality (HR = 2.31; 95 % CI:2.13**–**2.51, *P* < 0.0001). Model II reveals that for each 10-point increment in the FI, CVD mortality escalates by 82 % (HR =1.82; 95 % CI:1.52**–**2.19, *P* < 0.0001). All-cause mortality rose by 66 % (HR = 1.66; 95 % CI:1.51**–**1.83, *P* < 0.0001). Model III indicated a 54 % hike in CVD mortality for each 10-point increase in the FI (HR = 1.54; 95 % CI:1.24**–**1.91, *P* < 0.0001). There was a 55 % increase in all-cause mortality (HR = 1.55; 95 % CI:1.38**–**1.73, *P* < 0.0001) [[42], [43], [44]]. Kaplan-Meier analysis delineated significant disparities in both all-cause and CVD mortality between high-versus low-FI groups (Log-rank *P* < 0.001; refer to Supplementary Figure 2). This study highlights the FI as a risk factor for increased mortality, particularly related to cardiovascular outcomes and suggests that elevated FI could serve as prognostic indicators of unfavorable outcomes in CKM syndrome.

**Table 3:** Multivariable Cox regression analysis.

	Unadjusted		Adjust I		Adjust II	
	HR (95 %CI)	P	HR (95 %CI)	P	HR (95 %CI)	P
Cardiovascular mortality						
FI ≤0.1512	1.0		1.0		1.0	
FI > 0.1512	8.23 (5.02, 13.51)	<0.0001	3.70 (2.21, 6.19)	<0.0001	2.55 (1.46, 4.43)	0.0009
FI (per10 units)	2.58 (2.20, 3.03)	<0.0001	1.82 (1.52, 2.19)	<0.0001	1.54 (1.24, 1.91)	<0.0001
All-cause mortality						
FI ≤ 0.1512	1.0		1.0		1.0	
FI < 0.1512	5.42 (4.32, 6.80)	<0.0001	2.51 (1.98, 3.18)	<0.0001	2.06 (1.59, 2.66)	<0.0001
FI (per10 units)	2.31 (2.13, 2.51)	<0.0001	1.66 (1.51, 1.83)	<0.0001	1.55 (1.38, 1.73)	<0.0001

Abbreviations: HR, hazard ratio; CI, confidence interval; FI, Frailty index. Unadjusted model adjusted for: None.

Model I was adjusted for: Gender, Age, Race, Marital status categorized, PIR, Educational level, Alcohol consumption categorized, Smoking status.

Model II was adjusted for: Gender, Age, Race, Marital status categorized, PIR, Educational level, Smoking status, Alcohol consumption categorized, Scr, UA, TG, TC, HDL-C, LDL-C, Hypertension, Diabetes, Hyperlipidemia, PLT, eGFR, HGB.

### 3.4 Nonlinear relationships of FI with all-cause and CVD mortality

**Fig. 2:**
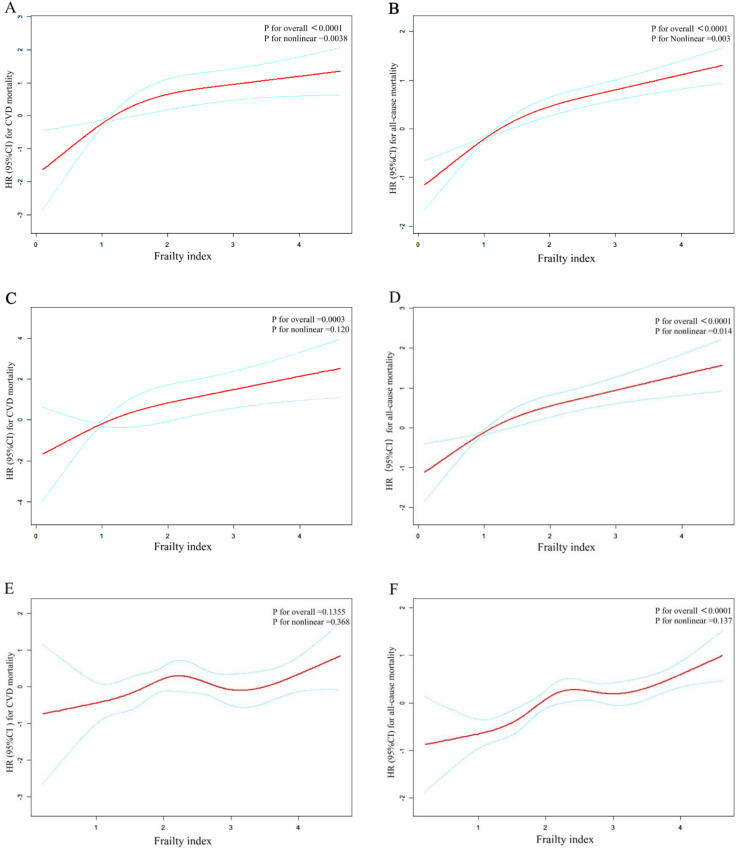
Smoothed Curve - Fitting Graph FIGURE 2 Nonlinear associations Frailty index and all-cause mortality, CVD mortality among CKM stage 0–2 and stage 3–4 populations A: The relationship between Frailty index and CVD mortality among CKM stage 0–4 populations (fully adjusted RCS Model). B: The relationship between Frailty index and all-cause mortality among CKM stage 0–4 populations (fully adjusted RCS Model). C: The relationship between Frailty index and CVD mortality among CKM stage 0–2 populations (fully adjusted RCS Model). D: The relationship between Frailty index and all-cause mortality among CKM stage 0–2 populations (fully adjusted RCS Model). E: The relationship between Frailty index and CVD mortality among CKM stage 3–4 populations (fully adjusted RCS Model). F:The relationship between Frailty index and all-cause mortality among CKM stage 3–4 populations (fully adjusted RCS Model). Adjustments in the model accounted for the following variables: Gender, Age, Race, Marital status categorized; PIR; Educational level; Alcohol consumption categorized; Smoking status; Scr; UA; TG; TC; HDL-C; LDL-C; Hypertension, Hyperlipidemia, Diabetes, PLT; eGFR; HGB.

Fig. 2 shows the results of the RCS analysis examining the nonlinear relationship between FI and the risk of all-cause and CVD mortality among patients with CKM syndrome, stratified by disease severity (early stages 0–2 compared with later phases 3–4). After full adjustment for covariates in Model III, a statistically significant nonlinear association was observed between FI and both all-cause mortality (*P* for nonlinearity < 0.05) and CVD mortality (*P* for nonlinearity < 0.05). A positive dose-response gradient was observed between higher FI values and higher risk of mortality. Threshold effect analyses based on dose-response curve fitting indicated threshold effects for all-cause mortality (FI = 2.34) and CVD mortality (FI = 2.18) (Table 4). Below these thresholds, each additional unit of FI was associated with a significant increase in the risk of all-cause mortality (adjusted HR: 2.09, 95 % CI: 1.65**–**2.64) and CVD mortality (adjusted HR: 2.61, 95 % CI: 1.49**–**4.57). However, beyond these inflection points, the associations weakened and did not reach statistical significance (HR = 1.22, 95 % CI: 1.00**–**1.48; *P* = 0.055 for all-cause mortality; HR = 1.18, 95 % CI: 0.84**–**1.65; *P* = 0.354 for CVD mortality). These findings delineate a critical threshold in the FI, above which the trajectory of mortality risk asymptotically stabilizes, underscoring the importance of early detection and preventive interventions in vulnerable populations with CKM syndrome.

**Table 4:** Threshold effect analysis of the FI and all-cause and cardiovascular mortality.

	cardiovascular mortality		all-cause mortality	
	HR (95 %CI)	P	HR (95 %CI)	P
Model I				
One line effect	1.54 (1.24, 1.91)	<0.0001	1.55 (1.38, 1.73)	<0.0001
Model II				
Turning point (K)	2.18		2.34	
FI< K	2.61 (1.49, 4.57)	0.0008	2.09 (1.65, 2.64)	<0.0001
FI> K	1.18 (0.84, 1.65)	0.3539	1.22 (1.00, 1.48)	0.0552
p value for LRT test*	0.038		0.003	

Abbreviations: HR, hazard ratio; CI, confidence interval.

Adjust: Gender, Age, Race, Marital status categorized; PIR; Educational level; Alcohol consumption categorized; Smoking status; Scr; UA; TG; TC; HDL-C; LDL-C; Hypertension, Hyperlipidemia, Diabetes, PLT; eGFR; HGB.

*P < 0.05 indicates that model II is significantly different from Model I.

### 3.5 The predictive performance of the FI for CKM syndrome, all-cause mortality and CVD mortality

**Fig. 3:**
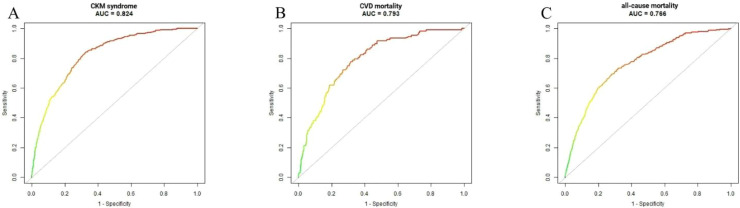
ROC curve analysis FIGURE 3 ROC curve analysis of Frailty index predicting CKM Stage 0 to 4, all-cause or cardiovascular mortality. The AUC for CKM syndrome, CVD and all-cause mortality were 0.824, 0.793, and 0.766, respectively.

The ROC curves, used to evaluate the predictive performance of the models, are presented in detail in Fig. 3 and Supplementary Table S3. The fully adjusted model for CKM syndrome yielded an AUC of 0.824 (95 % CI: 0.8097**–**0.8387), indicating that the model correctly discriminates between approximately 82.4 % of individuals with and without CKM syndrome. A specificity of 0.673 indicates that the model accurately identifies 67.3 % of those without the syndrome. The model achieved an AUC of 0.766 for predictin*g* all-cause mortality rates (95 % CI: 0.743**–**0.789). At the optimal cutoff point, sensitivity was 69.3 % and specificity was about 71.9 %. In the prediction of CVD mortality, the AUC increases to 0.793 (95 % CI: 0.754**–**0.832), with sensitivity reaching 77.8 % and specifically at 68.3 %.

### 3.6 Stratified associations between FI and all-cause and CVD mortality

**Fig. 4:**
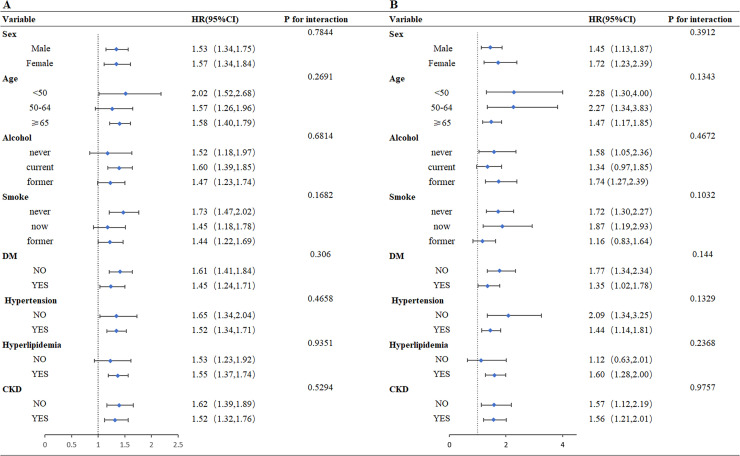
Relationship between frailty index and all-cause mortality and cardiovascular mortality in populations with CKM syndrome in different subgroups Abbreviations: HR, hazard ratio; CI, confidence interval; FI, Frailty index; CKM Syndrome, Cardiovascular-Kidney-Metabolic Syndrome DM, Diabetes mellitus; CKD, Chronic kidney disease; Smoke, Smoking status; Alcohol, Alcohol consumption categorized. Subgroup analyses were conducted to test the stability of the relationship between the Frailty index and the risk of all-cause mortality and CVD mortality. The primary outcome was particularly stable in all subgroups. A:FI with all-cause mortality, B:FI with cardiovascular mortality Adjust variables: Gender, Age, Race, Marital status categorized; PIR; Educational level; Alcohol consumption categorized; Smoking status; Scr; UA; TG; TC; HDL-C; LDL-C; Hyperlipidemia, Diabetes, PLT; eGFR; HGB.

The present study, based on nationally representative data, found that higher FI scores were significantly associated with an increased risk of all-cause and CVD mortality among patients with CKM syndrome. No statistically significant effect modification was observed across any of the tested subgroups. Fig. 4 shows stratified subgroup analyses of the association between FI thresholds and all-cause and CVD mortality, stratified by demographic and clinical factors, including age, sex, alcohol consumption, smoking status, diabetes, hypertension, and hyperlipidemia. These analyses were conducted to assess potential effect modification by these variables. Notably, no statistically significant interactions were observed between FI and any of the subgrouping variables (*P* for interaction >0.05 for all comparisons), indicating that the association between frailty and mortality remains consistent regardless of age, lifestyle factors, or cardiometabolic comorbidities.

## 4 Discussion

This research investigated the association between the FI and all-cause and CVD mortality in individuals with CKM syndrome. Using a nationally representative sample of 7049 adults from NHANES (2011–2018), we found that elevated FI values were independently associated with an increased risk of CKM syndrome. Multivariable piecewise Cox regression analysis revealed that higher FI levels were significantly associated with prevalent CKM syndrome (HR = 2.56). Among patients diagnosed with CKM syndrome, each 10-unit increment in FI was associated with a 54 % higher risk of CVD mortality, and 55 % higher risk of all-cause mortality. Kaplan-Meier survival curves consistently showed that the high-FI group had significantly poorer survival outcomes for both endpoints (log-rank *P* < 0.001). In terms of model discriminatory ability, models incorporating the FI demonstrated good predictive performance, with an AUC of 0.824 for CKM syndrome, 0.766 for all-cause mortality, and 0.793 for CVD mortality. Additionally, subgroup analyses revealed consistent associations between FI and both all-cause and CVD mortality, with no significant heterogeneity across strata defined by age, sex, alcohol use, hypertension, hyperlipidemia, CKD, and diabetes.

Frailty has received increasing attention as a key clinical marker of reduced physiological reserves and increased vulnerability to stressors. It represents a measure of biological aging, capturing the variation in biological age among individuals with the same chronological age. Numerous studies have consistently demonstrated association between frailty and elevated risks of mortality from CVD and a range of other conditions [29,45,46]. However, research on the association between frailty and mortality in patients with CKM syndrome remains limited. Therefore, we conducted the first study to investigate the relationship between FI and both all-cause and CVD mortality across the full spectrum of CKM syndrome stages (0–4). Our findings reveal a nonlinear relationship between FI and the risks of all-cause and CVD mortality in these patients, consistent with conclusions from prior studies. A study by You Fan et al. demonstrated that a frailty state is associated with a substantially increased risk of mortality from various causes, including cardiovascular disease, stroke, cancer, and other conditions, in both adults aged 65 years and older and younger individuals [47]. In accordance with these findings, the FI was incorporated as a continuous variable into the Cox proportional hazards model. The results showed that, after comprehensive adjustment for confounding variables, FI remained significantly and positively associated with all-cause mortality and CVD mortality among CKM patients. To further explore this association, RCS curves were used to model the dose-response relationship between FI and mortality outcomes. These analyses demonstrated a significant nonlinear association between the FI and both all-cause and CVD mortality across the entire CKM population (stages 0–4), as well as within both early-stage (0–2) and late-stage (3–4) subgroups (P for nonlinearity < 0.05). Consistent with findings from prior systematic reviews, frailty has been identified as a significant predictor for both all-cause and CVD mortality [[48], [49], [50]].

The underlying mechanisms by which lower FI levels, compared with higher levels, are associated with a more rapid increase in all causes and CVD mortality remain unclear. The multifaceted nature of frailty, which influences multiple aspects of human functioning and encompasses various pathophysiological pathways extensively addressed in the literature, could explain this observation [13,51]. The development of frailty involves shifts between states of dynamic equilibrium, characterized by progressive deterioration in cellular, molecular, and physiological integrity, until surpassing a critical threshold, leading to the emergence of clinically observable frailty [52,53]. The transition from lower to mild-to-moderate frailty may be associated with imbalances across multiple systems, including myocardial structure and function, glomerular filtration rate, vascular endothelial function, and metabolic homeostasis. At this stage, the clinical manifestations are presently nonspecific, although the physiological reserve has already sharply declined. This reduction has been proven to significantly diminish an individual's tolerance to acute hemodynamic fluctuations, arrhythmias, and ischemic events - transient conditions that greatly raise mortality risk even with a minor elevation in the frailty index (FI).

Furthermore, our findings indicated that the mortality rate among CKM stage 3 patients was higher than that observed in stage 4 patients. Several potential explanations may account for this seemingly contradictory observation. First, according to the American Heart Association (AHA) CKM staging classification, Stage 3 is defined by the presence of subclinical cardiovascular disease in the absence of overt clinical events [1,35]. Patients in this stage may be at a critical juncture in the progression of cardiovascular disease, characterized by active chronic low-grade inflammation, endothelial dysfunction, and unstable atherosclerotic plaques [31], all of which confer a high risk of sudden cardiac mortality. Patients who survive high-risk events and progress to Stage 4 may represent a more resilient subgroup, which introduces survivor bias that could partially explain the lower observed mortality rate among patients with CKM stage 4. Second, patients across different stages may differ in disease awareness and treatment adherence. Compared to those in earlier stages, stage 4 patients are more likely to receive intensified, guideline-recommended therapy, closer follow-up, and multidisciplinary care. At the same time, due to their greater overall disease burden, stage 4 patients are more experience incomplete data collection, inability to complete all examinations, or death before full evaluation. This may result in the exclusion of the sickest individuals from the analytic sample and consequently lead to an underestimation of mortality in this group. In addition, several variables recommended by the AHA for refining stages 3–4 such as cardiac biomarkers, cardiac imaging, atrial fibrillation, coronary angiography and peripheral artery disease-were not available in NHANES [54]. Misclassification of advanced CKM stages is therefore possible and may have attenuated the apparent risk in stage 4. These factors could collectively explain the slightly higher mortality observed in stage 3 CKM compared to stage 4 in this study.

The Kaplan-Meier survival curves illustrated the divergence of survival trajectories across FI strata, demonstrating a marked increase in both all-cause and CVD mortality in the high-FI group compared with the low-FI group. Notably, these separation between curves emerged early during the observational period and widened progressively, particularly in the all-cause mortality analysis. This divergence persisted throughout the study duration (log-rank *P* < 0.001). This suggests that CKM patients with increasing frailty levels followed a less favorable survival trajectory throughout the entire follow-up period. Further ROC analysis demonstrated that predictive models incorporating FI exhibited good discriminatory performance across all three outcomes: CKM syndrome, all-cause mortality and CVD mortality. Unlike traditional risk models, which primarily rely on indicators such as age, blood pressure, lipid levels, blood glucose and renal function and often fail to reflect patients' physiological reserve and multisystem impairment the inclusion of FI-a composite measure capturing the cumulative extent of multisystem health deficits, significantly improves risk stratification in this high-risk population. The divergence of the survival curves, as shown in the Kaplan-Meier curves and substantiated by the elevated AUC value, substantiates the pragmatic predictive efficacy of FI. The presence of CVD, CKD, and metabolic syndrome, all of which are chronic diseases associated with nutritional depletion, has been documented in patients with CKM [55,56]. It is hypothesized that frailty may share common biological pathways with CKD, including chronic inflammation, oxidative stress, and endothelial dysfunction [57,58]. Chronic low-grade inflammation, characterized by elevated proinflammatory cytokines such as IL-6 and TNF-α, promotes frailty development and accelerate atherosclerosis progression [59]. This process leads to impaired insulin sensitivity, abnormal glucose tolerance, disturbed cardiovascular system, renal injury, and hepatic steatosis. Ectopic fat has also been shown to trigger the secretion of local mediators that can induce organ damage, particularly in the heart and kidney. Such damage contributes to the development of several complications, including arrhythmias, myocardial infarction, coronary artery disease, and arterial hypertension. The latter involves increased oxidative stress associated with aging and increasing frailty, which directly damaging the vascular endothelium and activating lipid peroxidation. The process initiates the oxidation of LDL-C, leading to development of atherosclerotic plaques [60]. Secondly, malnutrition, a prevalent comorbidity in populations with frailty, exacerbates protein-energy depletion through impaired anabolic utilization and accelerated muscle catabolism. This process shifts nitrogen balance into the negative state, thereby promoting protein-energy wasting, sarcopenia, and loss of physiological reserve. It is well established that patients with elevated FI levels frequently present with several concomitant risk factors, including chronic inflammation, malnutrition, and reduced physical activity. These factors have been shown to accelerate glomerular and tubulointerstitial damage via inflammation and oxidative stress, thereby hastening renal decline. Concurrently, they induce protein-energy wasting (PEW), characterized by anorexia, decreased energy and protein consumption, and heightened muscle protein degradation due to inflammation, acidosis, and insulin resistance. In the uremic environment of chronic kidney disease (CKD), inflammatory mediators, oxidative stress, and uremic toxins inhibit muscle protein synthesis and promote catabolism, worsening protein-energy wasting (PEW) and muscle loss. Progressive renal dysfunction can be viewed as occurring simultaneously with the systemic depletion of protein reserves, creating a vicious cycle of renal deterioration, protein loss, and deteriorating frailty [[61], [62], [63]]. The combination of these factors culminates in a range of harmful impacts on an already compromised physiological state, notably amplifying the load on metabolic, cardiovascular, and renal systems. The intricate and synergistic effects of these multisystem complications are a crucial mechanism contributing to the elevated mortality linked with CKM syndrome, offering a pathophysiological rationale for its significant decrease in life expectancy. Our findings align with numerous previous studies showing that cardiovascular disease (CVD), diabetes, obesity, and renal impairment individually and collectively contribute to increased mortality risk [[64], [65], [66]]. Stratifying CKM patients by frailty severity holds potential for early identification of high-risk individuals and prognosis prediction. Clinicians can customize treatment strategies, conduct timely risk assessments, and monitor frailty index trends to facilitate prompt treatment adjustments and preventive interventions, maximizing opportunities to reduce mortality risk.

In subgroup analyses, we did not observe significant interactions between FI and the examined strata; all P values for interaction were greater than 0.05. In other words, the association between frailty and mortality was consistent across groups stratified by age and sex, as well as by the presence or absence of smoking, alcohol consumption, diabetes, hypertension, and hyperlipidemia. This consistency across diverse clinical and demographic subgroups supports the use of FI as a broadly generalizable prognostic marker in the CKM syndrome population. However, even after extensive adjustment for potential confounders, residual heterogeneity is likely to persist. CKM syndrome is inherently complex, and heterogeneity may remain despite the inclusion of a comprehensive set of relevant confounders. Patients may be classified into the same "subgroup" based on observable characteristics such as age or comorbidities, such as age or comorbidities, yet they may differ substantially with respect to unmeasured biological, clinical, or social factors. These latent differences in frailty and mortality risk could influence the strength or direction of the FI–mortality association in ways not fully captured by the current analyses. Therefore, although we did adjust for key variables including sex, age, hypertension, diabetes, CKD, dyslipidemia, smoking, and alcohol use, these are clearly imperfect proxies for the underlying residual heterogeneity. Moreover, unmeasured variability may attenuate or enhance the ability of FI to discriminate subgroups within our sample, even among individuals who appear similar demographically and clinically. This underscores the need for more detailed, mechanistic studies to elucidate how FI interacts with the disease processes of different CKM phenotypes. Understanding these interactions would enable further refinement of risk stratification and the development of targeted intervention strategies in this complex patient population.

## 5 Limitation

The primary limitation of this study is its retrospective, observational design, which limits our ability to establish firm causal inferences between the FI and mortality. Although statistically significant and strong associations were observed between high FI scores and both all-cause mortality and CVD mortality, these findings should be interpreted as patterns of association rather than evidence of causality. This distinction is particularly important given the potential clinical utility of FI as a prognostic tool. Further studies using prospective longitudinal designs are required to clarify temporal relationships and to determine whether changes in frailty status directly influence clinical outcomes. Such research may also help identify the key determinants of frailty progression. This type of research is necessary not only to validate existing evidence but also to uncover the biological mechanisms underlying the relationship between frailty and unfavorable outcomes, which may ultimately inform more targeted interventions and contribute to reducing mortality rates in patients with CKM syndrome. Another significant limitation stems from the potential for residual confounding due to variables that were not measured or fully captured. Although the analysis accounts for a broad range of variables, such as sex, age, BMI, and critical lab indices, it is not beyond the possibility that an important determinant of mortality has been eliminated. Waist circumference, blood urea nitrogen (BUN), B-type natriuretic peptide (BNP), troponin, detailed lifestyle factors, and relevant genetic markers are among the variables that could improve risk assessment for both frailty and mortality in this population. We applied advanced statistical methods, including multivariate Cox proportional hazards models, to control confounding factors as comprehensively as possible; however, unmeasured or inadequately measured variables cannot be eliminated. The findings suggest that any unknown factor needs to be associated with both FI and mortality to fully explain the observed associations, which further strengthens the robustness of our results despite residual uncertainty.

## 6 Conclusion

This study highlights the significant association between the FI and the incidence of both all-cause and CVD mortality in patients with CKM syndrome. Elevated FI is a strongly predicted CKM syndrome prevalence and subsequent mortality. Furthermore, the FI may serve as a novel indicator for staging CKM syndrome and guiding tailored therapeutic approaches.

### Data sharing statement

Data from the National Health and Nutrition Examination Survey (NHANES) are publicly available online.

## Funding

This work was supported by the Natural Science Foundation of Ningxia, China (NO. 2024AAC03561); Natural Science Foundation of Ningxia, China (NO. 2022AAC05058); Ningxia Health Commission Research Project(2025-NWZC-A003);Ningxia Medical University Intramural Research Project(XM2019049).

## CRediT authorship contribution statement

**Xin Wang:** Writing – original draft, Visualization, Validation, Software, Methodology, Investigation, Formal analysis, Data curation, Conceptualization. **Xinrui Hai:** Writing – original draft, Visualization, Validation, Software, Methodology, Investigation, Formal analysis, Data curation, Conceptualization. **Ali Ma:** Writing – original draft, Investigation. **Xiaolan Liang:** Writing – original draft, Investigation. **Hua Cheng:** Writing – review & editing, Supervision. **Peng Wu:** Writing – original draft, Investigation. **Yu Hao:** Writing – review & editing, Validation, Supervision, Investigation. **Dapeng Chen:** Writing – review & editing, Validation, Supervision, Investigation. **Ning Yan:** Writing – original draft, Validation, Supervision, Investigation.

## Declaration of competing interest

The authors declare that they have no known competing financial interests or personal relationships that could have appeared to influence the work reported in this paper.

## Appendix Supplementary materials

Image, application 1

## References

[1] Ndumele C.E., Rangaswami J., Chow S.L. (2023). Cardiovascular-kidney-metabolic Health: a presidential advisory from the American Heart Association [J]. Circulation.

[2] Kadowaki T., Maegawa H., Watada H. (2022). Interconnection between cardiovascular, renal and metabolic disorders: a narrative review with a focus on Japan [J]. Diabetes Obes Metab.

[3] Marassi M., Fadini G.P (2023). The cardio-renal-metabolic connection: a review of the evidence [J]. Cardiovasc Diabetol.

[4] SUH S., LEE M.K (2014). Metabolic syndrome and cardiovascular diseases in Korea [J]. J Atheroscler Thromb.

[5] Ahmad F B Anderson R N (2021). The leading causes of death in the US for 2020 [J]. Jama.

[6] Seferović P.M., Petrie M.C., Filippatos G.S. (2018). Type 2 diabetes mellitus and heart failure: a position statement from the Heart Failure Association of the European Society of Cardiology [J]. Eur J Heart Fail.

[7] Usman M.S., Khan M.S., Butler J. The interplay between diabetes, cardiovascular disease, and kidney disease [J]. 2021. 34279879

[8] Ostrominski J.W., Arnold S.V., butler J. (2023). Prevalence and overlap of cardiac, renal, and metabolic conditions in US adults, 1999-2020 [J]. JAMA Cardiol.

[9] Clegg A., Young J., Iliffe S. (2013). Frailty in elderly people [J]. Lancet.

[10] Hubbard R E Theou O (2012). Frailty: enhancing the known knowns [J]. Age Ageing.

[11] Rockwood K., Mitnitski A. (2011). Frailty defined by deficit accumulation and geriatric medicine defined by frailty [J]. Clin Geriatr Med.

[12] Mitnitski A.B., Mogilner A.J., Rockwood K. (2001). Accumulation of deficits as a proxy measure of aging [J]. Sci World J.

[13] Fried L.P., Tangen C.M., Walston J. (2001). Frailty in older adults: evidence for a phenotype [J]. J Gerontol Biol Sci Med Sci.

[14] Blodgett J.M., Theou O., Howlett S.E. (2017). A frailty index from common clinical and laboratory tests predicts increased risk of death across the life course [J]. Geroscience.

[15] Adabag S., Vo T.N., Langsetmo L. (2018). Frailty as a risk factor for cardiovascular versus noncardiovascular mortality in older men: results from the MrOS sleep (outcomes of sleep disorders in older men) study [J]. J Am Heart Assoc.

[16] Muscedere J., Waters B., Varambally A. (2017). The impact of frailty on intensive care unit outcomes: a systematic review and meta-analysis [J]. Intensive Care Med.

[17] Aguayo G.A., Vaillant M.T., Donneau A.F. (2018). Comparative analysis of the association between 35 frailty scores and cardiovascular events, cancer, and total mortality in an elderly general population in England: an observational study [J]. PLoS Med.

[18] Wallace L.M., Theou O., Kirkland S.A. (2014). Accumulation of non-traditional risk factors for coronary heart disease is associated with incident coronary heart disease hospitalization and death [J]. PLoS One.

[19] Farooqi M.A.M., Gerstein H., Yusuf S. (2020). Accumulation of deficits as a key risk factor for cardiovascular morbidity and mortality: a pooled analysis of 154 000 individuals [J]. J Am Heart Assoc.

[20] Blodgett J., Theou O., Kirkland S. (2015). The association between sedentary behaviour, moderate-vigorous physical activity and frailty in NHANES cohorts [J]. Maturitas.

[21] Kehler D.S., Ferguson T., Stammers A.N. (2017). Prevalence of frailty in Canadians 18-79 years old in the Canadian health measures survey [J]. BMC Geriatr.

[22] García-Esquinas E., José García-García F., León-muñoz L.M. (2015). Obesity, fat distribution, and risk of frailty in two population-based cohorts of older adults in Spain [J]. Obes (Silver Spring).

[23] León-Muñoz L.M., García-Esquinas E., López-García E. (2015). Major dietary patterns and risk of frailty in older adults: a prospective cohort study [J]. BMC Med.

[24] Morley J.E., Malmstrom T.K., Rodriguez-Mañas L. (2014). Frailty, sarcopenia and diabetes [J]. J Am Med Dir Assoc.

[25] Ramsay S.E., Arianayagam D.S., Whincup P.H. (2015). Cardiovascular risk profile and frailty in a population-based study of older British men [J]. Heart.

[26] Woods N.F., Lacroix A.Z., Gray S.L. (2005). Frailty: emergence and consequences in women aged 65 and older in the Women's Health Initiative Observational Study [J]. J Am Geriatr Soc.

[27] Graciani A., GARCÍA-Esquinas E., López-García E. (2016). Ideal cardiovascular health and risk of frailty in older adults [J]. Circ Cardiovasc Qual Outcomes.

[28] Pandey A., Segar M.W., Singh S. (2022). Frailty status modifies the efficacy of exercise training among patients with chronic heart failure and reduced ejection fraction: an analysis from the HF-ACTION trial [J]. Circulation.

[29] Cao X., Yang Z., Li X. (2023). Association of frailty with the incidence risk of cardiovascular disease and type 2 diabetes mellitus in long-term cancer survivors: a prospective cohort study [J]. BMC Med.

[30] Larkin H. (2023). Here's what to know about cardiovascular-kidney-metabolic syndrome, newly defined by the AHA [J]. Jama.

[31] Ndumele C.E., Neeland I.J., Tuttle K.R. (2023). A synopsis of the evidence for the science and clinical management of cardiovascular-kidney-metabolic (CKM) syndrome: a scientific statement from the American heart association [J]. Circulation.

[32] Searle S.D., Mitnitski A., Gahbauer E.A. (2008). A standard procedure for creating a frailty index [J]. BMC Geriatr.

[33] Hakeem F.F., BERNABÉ E., Sabbah W. (2021). Association between oral health and frailty among American older adults [J]. J Am Med Dir Assoc.

[34] Pang S., Miao G., Zhou Y. (2023). Association between coffee intake and frailty among older American adults: a population-based cross-sectional study [J]. Front Nutr.

[35] Aggarwal R., Ostrominski J.W., Vaduganathan M. (2024). Prevalence of cardiovascular-kidney-metabolic syndrome stages in US adults, 2011-2020 [J]. Jama.

[36] Khan S.S., Matsushita K., Sang Y. (2024). Development and validation of the American heart association's PREVENT equations [J]. Circulation.

[37] KDIGO (2024). 2024 Clinical practice guideline for the evaluation and management of chronic kidney disease [J]. Kidney Int.

[38] Inker L.A., Eneanya N.D., Coresh J. (2021). New creatinine- and cystatin C-based equations to estimate GFR without race [J]. N Engl J Med.

[39] Dang K., Wang X., Hu J. (2024). The association between triglyceride-glucose index and its combination with obesity indicators and cardiovascular disease: NHANES 2003-2018 [J]. Cardiovasc Diabetol.

[40] Tang J., Xu Z., Ren L. (2024). Association of serum Klotho with the severity and mortality among adults with cardiovascular-kidney-metabolic syndrome [J]. Lipids Health Dis.

[41] Vandenbroucke J.P., Von E.LM.E., Altman D.G. (2007). Strengthening the reporting of Observational studies in epidemiology (STROBE): explanation and elaboration [J]. PLoS Med.

[42] Li W., Shen C., Kong W. (2024). Association between the triglyceride glucose-body mass index and future cardiovascular disease risk in a population with Cardiovascular-Kidney-Metabolic syndrome stage 0-3: a nationwide prospective cohort study [J]. Cardiovasc Diabetol.

[43] Moore M.K., Jones G.T., Mccormick S. (2024). Association between lipoprotein(a), LPA genetic risk score, aortic valve disease, and subsequent major adverse cardiovascular events [J]. Eur J Prev Cardiol.

[44] Zhang L., Sun H., Yin J. (2024). Association between triglyceride glucose-body mass index and depression among US adults: a cross-sectional study [J]. Public Health.

[45] Court T., Capkova N., Pająk A. (2024). Frailty index is an independent predictor of all-cause and cardiovascular mortality in Eastern Europe: a multicentre cohort study [J]. J Epidemiol Community Health.

[46] He D., Wang Z., Li J. (2024). Changes in frailty and incident cardiovascular disease in three prospective cohorts [J]. Eur Heart J.

[47] Fan J., Yu C., Guo Y. (2020). Frailty index and all-cause and cause-specific mortality in Chinese adults: a prospective cohort study [J]. Lancet Public Health.

[48] Chang S.F., Lin P.L (2015). Frail phenotype and mortality prediction: a systematic review and meta-analysis of prospective cohort studies [J]. Int J Nurs Stud.

[49] Kane R.L., Shamliyan T., Talley K. (2012). The association between geriatric syndromes and survival [J]. J Am Geriatr Soc.

[50] Shamliyan T., Talley K.M., Ramakrishnan R. (2013). Association of frailty with survival: a systematic literature review [J]. Ageing Res Rev.

[51] Rockwood K., Song X., Macknight C. (2005). A global clinical measure of fitness and frailty in elderly people [J]. Cmaj.

[52] Trevisan C., Veronese N., Maggi S. (2017). Factors influencing transitions between frailty states in elderly adults: the Progetto Veneto Anziani longitudinal Study [J]. J Am Geriatr Soc.

[53] Xue Q.L (2011). The frailty syndrome: definition and natural history [J]. Clin Geriatr Med.

[54] Zhu R., Wang R., He J. (2025). Associations of cardiovascular-kidney-metabolic syndrome stages with premature mortality and the role of social determinants of health [J]. J Nutr Health Aging.

[55] Lv J., Li R., Yuan L. (2022). Research on the frailty status and adverse outcomes of elderly patients with multimorbidity [J]. BMC Geriatr.

[56] Tong X., Xu J., Gong E. (2024). Frailty as a breakthrough point for multimorbidity management among older adults: challenges and opportunities in China [J]. Bmj.

[57] Afilalo J., Alexander K.P., Mack M.J. (2014). Frailty assessment in the cardiovascular care of older adults [J]. J Am Coll Cardiol.

[58] Fleg J.L., Aronow W.S., Frishman W.H (2011). Cardiovascular drug therapy in the elderly: benefits and challenges [J]. Nat Rev Cardiol.

[59] Furman D., Campisi J., Verdin E. (2019). Chronic inflammation in the etiology of disease across the life span [J]. Nat Med.

[60] Gioscia-Ryan R.A., Battson M.L., Cuevas L.M. (2016). Voluntary aerobic exercise increases arterial resilience and mitochondrial health with aging in mice [J]. Aging (Albany NY).

[61] Nixon A.C., Bampouras T.M., Pendleton N. (2018). Frailty and chronic kidney disease: current evidence and continuing uncertainties [J]. Clin Kidney J.

[62] Chang J., Liang Y., Sun P. (2025). Molecular and cellular mechanisms linking chronic kidney disease and sarcopenia in aging: an integrated perspective [J]. Clin Interv Aging.

[63] Tsai C.C., Wang P.C., Hsiung T. (2025). Sarcopenia in chronic kidney disease: a narrative review from pathophysiology to therapeutic approaches [J]. Biomedicines.

[64] Li Y., Ning Y., Shen B. (2023). Temporal trends in prevalence and mortality for chronic kidney disease in China from 1990 to 2019: an analysis of the Global Burden of Disease Study 2019 [J]. Clin Kidney J.

[65] Roth G.A., Mensah G.A., Johnson C.O. (2020). Global burden of cardiovascular diseases and Risk factors, 1990-2019: update from the GBD 2019 study [J]. J Am Coll Cardiol.

[66] Taylor K.S., Heneghan C.J., Farmer A.J. (2013). All-cause and cardiovascular mortality in middle-aged people with type 2 diabetes compared with people without diabetes in a large U.K. primary care database [J]. Diabetes Care.

