# Supplementary material for: Association between the frailty index and all-cause and cardiovascular mortality in a population with cardiovascular-kidney-metabolic syndrome: Insights from the NHANES 2011-2018
Source: J Frailty Aging. 2026 Jan 29;15(2):100131. doi: 10.1016/j.tjfa.2025.100131 (PMC12873725; doi:10.1016/j.tjfa.2025.100131)
Supplement: Supplementary file 1 [file mmc1.docx]

**SUPPLEMENTAL MATERIAL**

**Supplementary Table S1. The basic PREVENT 10-year risk estimation model equations**

| **10-year CVD risk assessment equation** | |
| --- | --- |
| **Men** | **log-Odds** = -3.031168 + 0.7688528 × (age – 55) /10 + 0.0736174 × ((TC – HDL-C) × 0.02586 – 3.5) – 0.0954431 × (HDL-C × 0.02586 – 1.3) /0.3 – 0.4347345 × (min(SBP, 110) – 110) /20 + 0.3362658 × (max(SBP, 110) – 130) /20 + 0.7692857 × (if diabetes) + 0.4386871 × (if current smoker) + 0.5378979 × (min(eGFR, 60) – 60) / -15 + 0.0164827 × (max(eGFR, 60) – 90) / -15 + 0.288879 × (if using anti hypertensive medication) – 0.1337349 × (if using statin) – 0.0475924 × (if using anti-hypertensive medication) × (max(SBP, 110) – 130) /20 + 0.150273 × (if using statin) × ((TC – HDL-C) × 0.02586 – 3.5) – 0.0517874 × (age – 55) /10 × ((TC – HDL-C) × 0.02586 – 3.5) + 0.0191169 × (age – 55) /10 × (HDL-C × 0.02586 – 1.3) /0.3 – 0.1049477 × (age – 55) /10 × (max(SBP, 110) – 130) /20 – 0.2251948 × (age – 55) /10 × (if diabetes) – 0.0895067 × (age – 55) /10 × (if current smoker) – 0.1543702 × (age – 55) /10 × (min(eGFR, 60) – 60) / -15 |
|  | **Risk**= 1 / (1 + exp(-log-Odds)) |
| **Women** | log-Odds = -3.307728 + 0.7939329 × (age – 55) /10 + 0.0305239 × ((TC – HDL-C) × 0.02586 – 3.5) – 0.1606857 × (HDL-C × 0.02586 – 1.3) /0.3 – 0.2394003 × (min(SBP, 110) – 110) /20 + 0.360078 × (max(SBP, 110) – 130) /20 + 0.8667604 × (if diabetes) + 0.5360739 × (if current smoker) + 0.6045917 × (min(eGFR, 60) – 60) / -15 + 0.0433769 × (max(eGFR, 60) – 90) / -15 + 0.3151672 × (if using anti hypertensive medication) – 0.1477655 × (if using statin) – 0.0663612 × (if using anti-hypertensive medication) × (max(SBP, 110) – 130) /20 + 0.1197879 × (if using statin) × ((TC – HDL-C) × 0.02586 – 3.5) – 0.0819715 × (age – 55) /10 × ((TC – HDL-C) × 0.02586 – 3.5) + 0.0306769 × (age – 55) /10 × (HDL-C × 0.02586 × 0.02586 – 1.3) /0.3 – 0.0946348 × (age – 55) /10 × (max(SBP, 110) – 130) /20 – 0.27057 × (age – 55) /10 × (if diabetes) – 0.078715 × (age – 55) /10 × (if current smoker) – 0.1637806 × (age – 55) /10 × (min(eGFR, 60) – 60) / -15 |
|  | **Risk**= 1 / (1 + exp(-log-Odds)) |

Abbreviations: TC, total cholesterol; HDL-C, high-density lipoprotein cholesterol; SBP, systolic blood pressure; eGFR, estimated glomerular filtration rate.

**Supplementary Table S2 Methods for evaluating each CKM stage**

| CKM health stages | Definition |
| --- | --- |
| Stage 0: No CKM health risk factors | Individuals with normal BMI and waist circumference, normoglycemia, normotension, a normal lipid profile, and no evidence of CKD or subclinical or clinical CVD.  Predicted 10-year CVD risk < 20%. |
| Stage 1: Excess and/or dysfunctional adiposity | Individuals with overweight/obesity, abdominal obesity, or dysfunctional adipose tissue, without the presence of other metabolic risk factors or CKD.  Predicted 10-year CVD risk < 20%. |
| Stage 2: Metabolic risk factors and CKD | Individuals with metabolic risk factors (hypertriglyceridemia, hypertension, MetS, diabetes), or CKD  Predicted 10-year CVD risk < 20%. |
| Stage 3: Subclinical CVD in CKM | Subclinical CVD among individuals with excess/dysfunctional adiposity, other metabolic risk factors, or CKD  Predicted 10-year CVD risk ≥ 20%. |
| Stage 4: Clinical CVD in CKM | Clinical CVD among individuals with excess/dysfunctional adiposity,  other metabolic risk factors, or CKD |

Abbreviations: BMI, body mass index; CKD, chronic kidney disease; CKM, cardiovascular-kidney-metabolic; CVD, cardiovascular disease

**Supplementary Table S3 Definitions of covariates and comorbidities.**

| **Category / Variable** | **Definition / Criteria** |
| --- | --- |
| **Demographic and Socioeconomic Factors** |  |
| Age | Measured continuously, with the actual age of each participant recorded. |
| Gender | Recorded as male or female. |
| Race/Ethnicity | Categorized as one of the following: non-Hispanic white, non-Hispanic black, Mexican American, or other races. |
| Poverty Income Ratio (PIR) | Employed as a continuous variable. |
| Educational Attainment | Divided into pre-middle school, secondary or equivalent, and post-secondary levels. |
| **Lifestyle and Anthropometric Factors** |  |
| Body Mass Index (BMI) | Determined by dividing weight in kg by the square of height in (kg/m²). |
| Smoking Status | **Never smokers:** Cumulative smoking <100 cigarettes and no history of regular smoking.  **Former smokers:** Cumulative smoking ≥100 cigarettes but have quit smoking completely for ≥6 months.  **Current smokers:** Cumulative smoking ≥100 cigarettes with regular (daily) or intermittent (non-daily) smoking behavior in the 30 days prior to the survey time point. |
| Alcohol Consumption Status | **Never drinkers:** Lifetime alcohol intake <12 standard drinking units and no history of regular alcohol consumption.  **Former drinkers:** Cumulative alcohol intake ≥ 12 standardized units, but no alcohol consumption in the past 12 months.  **Current drinkers:** Drinking behavior of any frequency in the past 12 months (including episodic or regular drinking). |
| **Clinical Comorbidities** |  |
| Hypertension | Classified as meeting any of these criteria:(1) Average Systolic BP of 140 mmHg or more and/or Diastolic BP at 90 mmHg or above, based on standardized measurements (after 5 minutes rest, averaged from multiple readings taken at least 2 minutes apart); (2) Self-reported history of hypertension confirmed by a healthcare provider;(3) Current use of antihypertensive medications. |
| Diabetes Mellitus | Identified upon satisfying any of the following conditions:(1) Glycated hemoglobin (HbA1c) ≥ 6.5%;(2) Fasting glucose ≥ 7.0 mmol/L (fasting time ≥ 8 hours); (3) Random plasma glucose or OGTT 2-hour glucose ≥11.1 mmol/L;(4) History of diabetes mellitus diagnosed by a licensed physician;(5) Current hypoglycemic treatments (oral agents or insulin-based). |
| Hyperlipidemia | Diagnosed based on meeting any of the subsequent standards:(1) Current use of statin, fibrate or other lipid-lowering drugs;(2) Triglycerides (TG): ≥150 mg/dL (1.7 mmol/L) (fasting ≥12 hours);(3) Total cholesterol (TC): ≥200 mg/dL (5.2 mmol/L);(4) Low-density lipoprotein cholesterol (LDL-C): ≥130 mg/dL (3.4 mmol/L);(5) High-density lipoprotein cholesterol (HDL-C): <40 mg/dL (1.0 mmol/L) (men) / <50 mg/dL (1.3 mmol/L) (women). |

**Supplementary Figure 1 Nonlinear relationships of Frailty index with CKM syndrome**

| 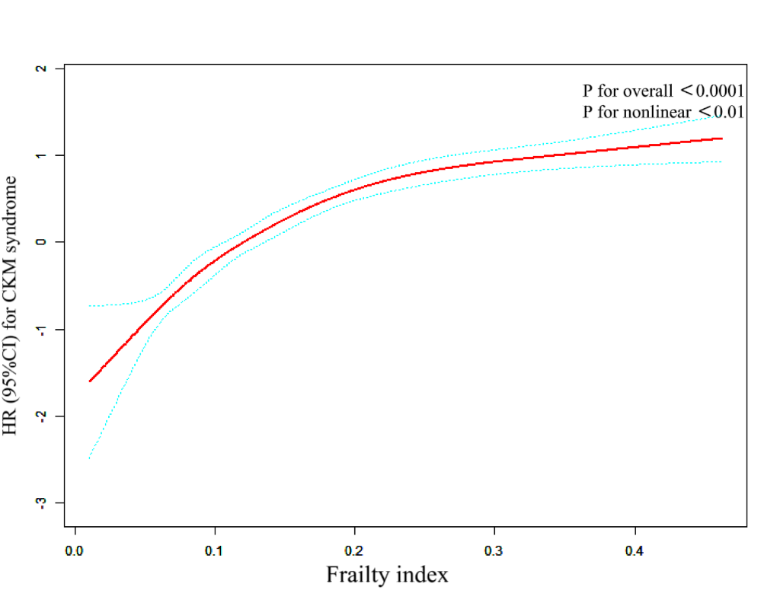 |
| --- |
| Supplementary Figure 1 Nonlinear relationships of Frailty index with CKM syndrome  Abbreviation: CKM syndrome Cardiovascular- Kidney- Metabolic Syndrome |

**Supplementary Figure 2** Kaplan–Meier curves for all-cause mortality (A), cardiovascular mortality(B).

| 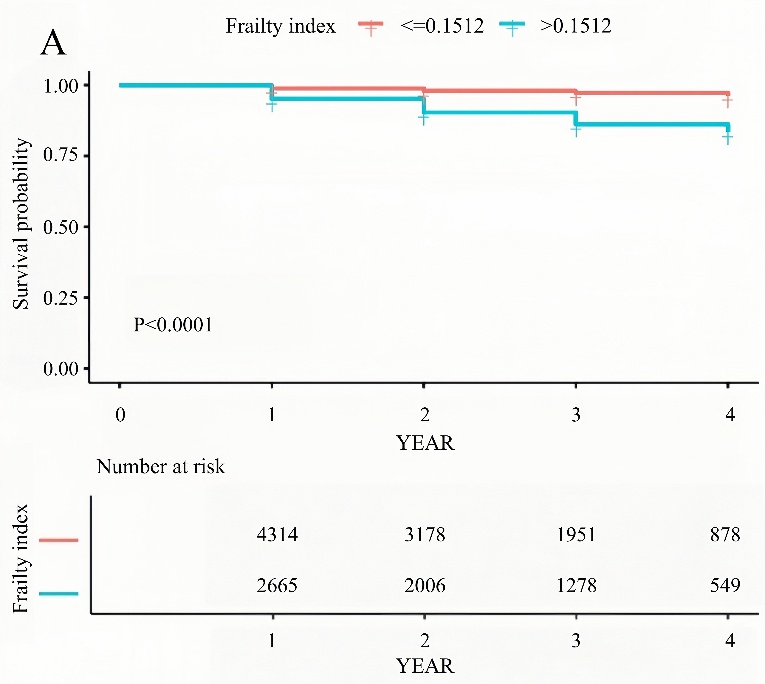 | 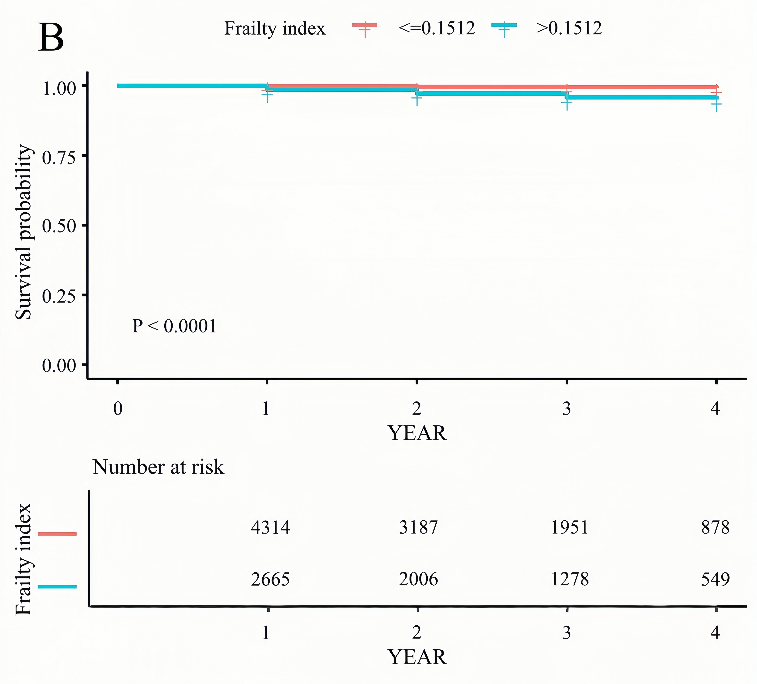 |
| --- | --- |
| **Supplementary Figure 2** Kaplan–Meier curves for all-cause mortality (A), cardiovascular mortality(B). | |

**Supplementary Table S3 The values of Receiver operating characteristic (ROC) curves of CKM Stages 0 to 4, all-cause and cardiovascular mortality**

| character | AUC (95%CI) | Specificity | Sensitivity |
| --- | --- | --- | --- |
|  |  |  |  |
| CKM | 0.8242(0.8097,0.8387) | 0.6727 | 0.8425 |
| all-cause mortality | 0.7658(0.7427,0.7888) | 0.7194 | 0.6931 |
| CVD mortality | 0.7930(0.7543,0.8317) | 0.6825 | 0.7778 |

Abbreviations: CKM, cardiovascular-kidney-metabolic; CVD, cardiovascular disease
